# Posterior axilla sling traction: a new technique for severe shoulder dystocia

**DOI:** 10.1136/bcr-2018-226882

**Published:** 2019-03-20

**Authors:** Jeffrey Hoek, Babette Verkouteren, Dennis van Hamont

**Affiliations:** 1 Department of Obstetrics and Gynaecology, Amphia Hospital, Breda, The Netherlands; 2 Department of Dermatology, Amphia Hospital, Breda, The Netherlands

**Keywords:** obstetrics and gynaecology, pregnancy

## Abstract

We describe a case of severe shoulder dystocia where, after failing of the known techniques, the posterior axilla sling traction technique was applied successfully. This technique was first described in 2009 by Hofmeyr and Cluver and must be considered at severe cases of shoulder dystocia where all other non-invasive techniques have failed.

## Background 

Shoulder dystocia is an unpredictable obstetric complication with possible severe damage to the new-born as well as the mother. Although some risk factors, for example, maternal diabetes, obesity and macrosomia have been identified, shoulder dystocia is still hard to predict. A broad spectrum of manoeuvres has been described over the years, which are all empirically applied.

Shoulder dystocia is a state during childbirth in which the shoulders are too wide to pass through the pelvis and the anterior shoulder of the fetus cannot pass below the pubic symphysis. The official definition is as follows: ‘a vaginal cephalic delivery that requires additional obstetric manoeuvres to deliver the fetus after the head has been delivered and gentle traction failed’.^1^

According to the Managing Obstetric Emergencies and Trauma (MOET) course, manoeuvres need to be applied in according to the acronym HELPERR (table 1). If all these methods fail, there are a few invasive manoeuvres left. These invasive manoeuvres are intentional breaking of the clavicle (cleidotomy), the Zavanelli manoeuvre followed by an immediate caesarean section or a symphysiotomy. All of these manoeuvres are associated with severe neonatal and maternal morbidity and are often not as easily performed as described in literature.

**Table 1:** The acronym HELPERR

H	Help
E	Episiotomy
L	Legs (‘McRoberts manoeuvre’)
P	Pressure suprapubic
E	Enter manoeuvres (‘Robin and Wood manoeuvre’)
R	Remove posterior arm
R	Roll the patient

In 2009, a new technique was described by Cluver and Hofmeyr.^2^ With this technique, a neonatal suction tube is placed under the posterior axilla as a sling, to which traction is applied. They originally tested it on cases where the fetus already died in utero after known methods for shoulder dystocia were unsuccessful. Because of the rapid success they started to practice the technique on several cases with severe shoulder dystocia before intrauterine death occurred.^3^ In response to this, a case in Switzerland had been described where applying traction to the sling alone was not successful.^4^ However, rotation of the posterior shoulder using the sling resulted in a quick delivery.

We think it is important for all gynaecologists and midwives to know about this minimal invasive, but effective, method.

## Case presentation

A 35-year-old woman, gravida 2 para 1, was admitted to our inpatient clinic for a vaginal birth during her second pregnancy.

In her obstetric history, she had undergone a secondary caesarean section due to a prolonged second stage of labour due to high birth weight (GA: 41 weeks and 3 days, 4130 g, p80–84) and a cephalic presentation in the occiput posterior position. In her current pregnancy, there was elaborate counselling regarding the delivery mode, she opted for a vaginal birth after caesarean section. Her pregnancy course and clinical examination were completely normal, except a fetal abdominal circumference above p99. We therefore decided to induce labour at 39+2 weeks of gestation.

At the day of delivery, she had a cervical dilatation of 4 cm and we artificially ruptured the membranes at 08:00. At 08:45, oxytocin was started to stimulate uterine contractions. It was difficult to stimulate the uterus, but on high dose oxytocin the patient eventually reached active phase of labour. At 17:10, she had a pain relief request due to severe labour pains. At that time, her cervix was 8 cm dilated and we decided to start with patient-controlled remifentanil. At 21:10, she reached full cervical dilation and she started second stage of labour while the caput of the fetus was just above the Hodge plane 3. Twenty minutes into the secondary stage of labour, the cardiotocography showed a fetal tachycardia and complicated, but variable, decelerations. At that moment, the fetal caput was at Hodge 4. Because there was rapid progression in the delivery an episiotomy in combination with fundal expression was applied. Directly after these manoeuvres, at 21:48, the fetal head was easily delivered with external rotation. This was immediately followed by a turtle sign. We unsuccessfully applied the McRoberts’ manoeuvre with suprapubic pressure followed by an also unsuccessful Woods corkscrew manoeuvre. Then, the Rubin II on all four was applied, which also failed. As a last step, we tried to manually deliver the posterior shoulder by traction on that posterior axilla; however, this technique also failed. In the moment before we moved on performing a symphysiotomy, we decided to try the posterior axilla sling traction technique. At this time the patient was still in the all fours position.

At 21:50, a neonatal suction tube was fed around the posterior axilla without any problems, creating a sling around the posterior shoulder. Thereafter, we applied gentle vertical traction on the suction tube and as a result the posterior shoulder was immediately born.

## Outcome and follow-up

At 21:51, the neonate was born with an Apgar-score of 2-8-9 and weighed 4010 g (p90–95). The neonate needed positive pressure ventilation during the first 2 min. Subsequently, the neonate stayed dependent on oxygen and the first days were complicated by an infection. The neonate recovered quickly with antibiotics and was dismissed from the hospital after 8 days. There were no signs of fractures, (central) nerve injuries or soft tissue injuries.

The patient had an episiotomy, two vaginal wall lacerations and a second-degree perineal laceration contralateral of the episiotomy. The lacerations and the episiotomy were sutured under local anaesthesia. Furthermore, there was a postpartum haemorrhage with a total blood loss of 1300 cc, mainly coming from the episiotomy. Unfortunately, the puerperium was complicated by endometritis which was adequately treated using antibiotics.

## Discussion

**Figure 1:**
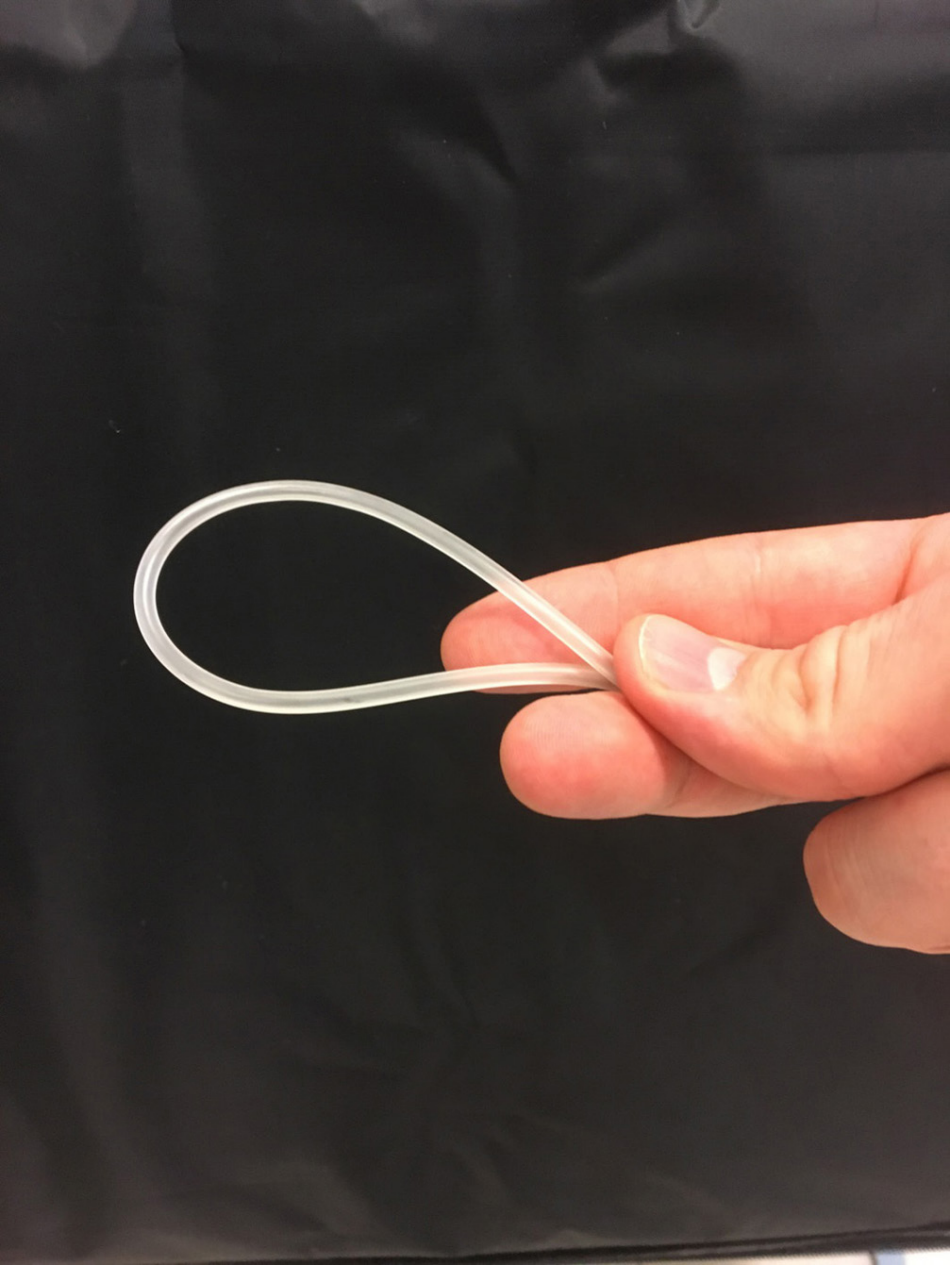
A neonatal suction tube is folded and hold together using your thumb and index finger.

**Figure 2:**
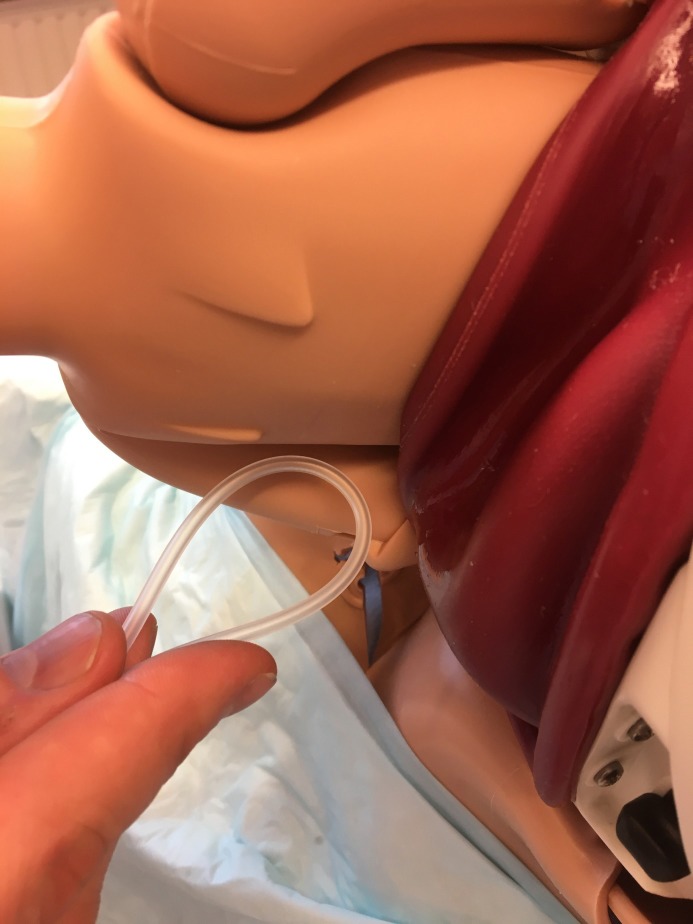
The suction tube is positioned under the posterior axilla of the neonate.

**Figure 3:**
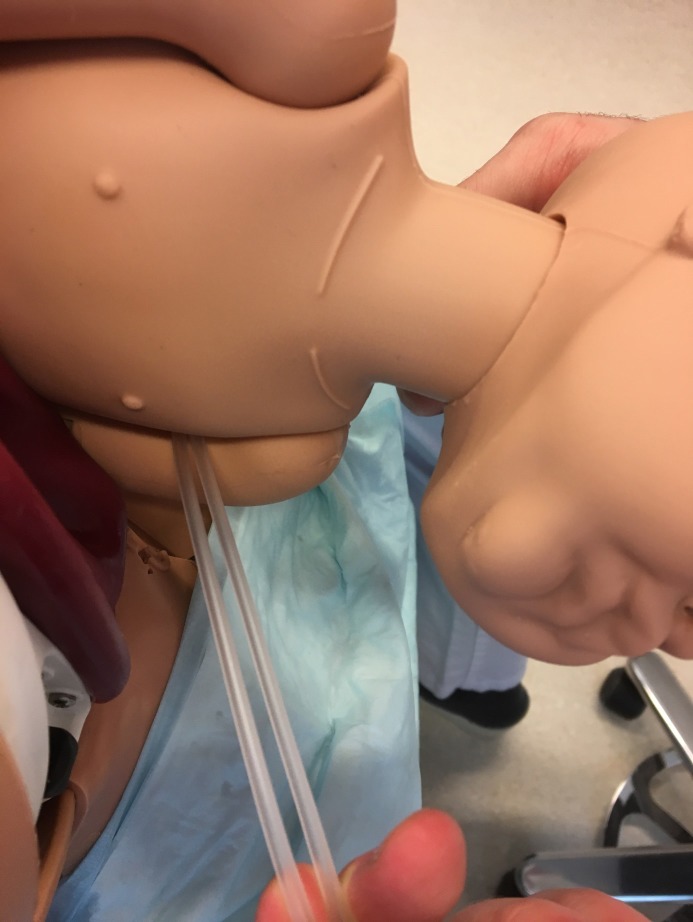
With the fingers of the other hand, the suction tube is caught on the other side of the armpit and pulled underneath.

**Figure 4:**
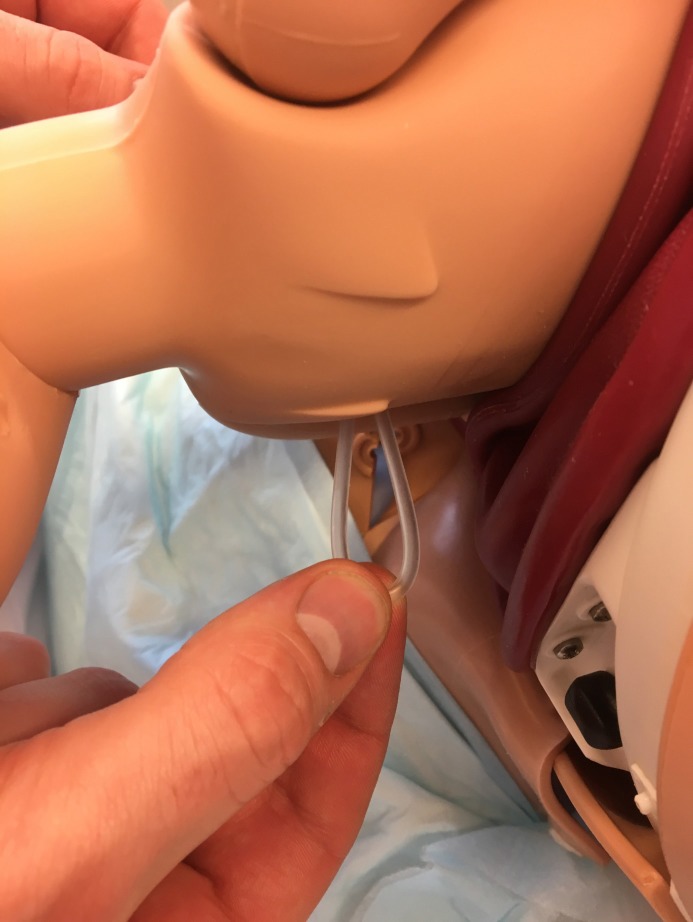
Opposite view of figure 3.

**Figure 5:**
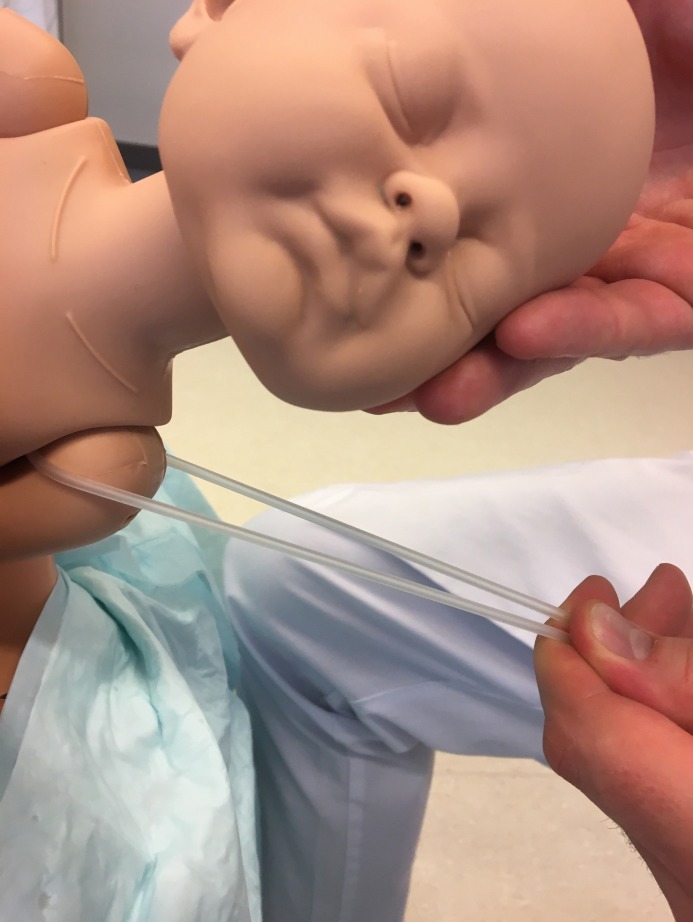
A sling is formed to which traction is applied.

As stated before, a case of shoulder dystocia has to be approached according to the MOET guidelines using the HELPERR acronym. All conventional methods according to HELPERR were unsuccessful in this case. Before moving on to invasive manoeuvres, we decided to apply the posterior axilla sling traction technique. With this technique a neonatal suction tube is folded and positioned under the posterior axilla of the neonate using your thumb and index finger (figures 1 and 2). With the fingers of the other hand, the suction tube is caught on the other side of the armpit and pulled underneath (figure 3). Then, a sling is formed to which traction is applied (figures 4 and 5). This will lead to the birth of the posterior arm and the rest of the foetus will follow quickly.

The technique of the posterior axilla sling traction is based on the digital hitching on the posterior axilla of the neonate, described in 2006 by Menticoglou.^2 5^

The advantage of the sling compared with the digital hitching of the posterior shoulder is the less needed space in the already narrow birth canal. With this technique, the operator can also apply more power due to a good grip on the sling. One can imagine that the use of a sling will also leave less damage to the birth canal compared with using your own hand.

Some case reports show the use of arterial forceps to hold the sling together.^3^ This ensures an even better grip on the sling. In the case report of Taddei *et al*, where the traction was not successful, the sling was eventually used to rotate the posterior shoulder.^4^ This option can be considered if traction alone fails to deliver the posterior shoulder. In most cases, a neonatal suction tube is used as sling material.^3 4^ In the other cases, a Foley catheter was used, which was believed to be too elastic.^3^

Cases of severe shoulder dystocia are rare and always emergency situations. It is impossible to evaluate and investigate this technique in large prospective studies. This and other cases show that the posterior axilla sling traction technique is an effective and relatively non-invasive method to resolve a severe shoulder dystocia. A neonatal suction tube is present in all western world neonatal resuscitation rooms. Furthermore, the technique is easily taught and learnt. Therefore, we believe that the posterior axilla sling traction technique must be included in the MOET-course. Thus, when a shoulder dystocia occurs during labour and the manoeuvres according to HELPERR did not resolve in childbirth, the posterior axilla sling technique must be considered before moving on to an invasive manoeuvre.

**Learning points**

- The posterior axilla sling traction technique is an effective and non-invasive method to resolve a severe shoulder dystocia.
- This technique is easily taught and learnt by obstetricians and midwives.
- This technique should be included in all obstetric emergency courses.
